# Effects of siRNA-mediated knockdown of jumonji domain containing 2A on proliferation, migration and invasion of the human breast cancer cell line MCF-7

**DOI:** 10.3892/etm.2012.662

**Published:** 2012-08-13

**Authors:** BEI-XU LI, CHENG-LIANG LUO, HUI LI, PENG YANG, MING-CHANG ZHANG, HONG-MEI XU, HONG-FEI XU, YI-WEN SHEN, AI-MIN XUE, ZI-QIN ZHAO

**Affiliations:** Department of Forensic Medicine, Shanghai Medical College, Fudan University, Shanghai 200032, P.R. China

**Keywords:** jumonji domain containing 2A, transfection, invasion, proliferation, migration

## Abstract

Jumonji domain containing 2A (JMJD2A) is a potential cancer-associated gene that may be involved in human breast cancer. The present study aimed to investigate suppressive effects on the MCF-7 human breast cancer cell line by transfection with JMJD2A-specific siRNA. Quantitative real-time PCR and western blot analysis were used to detect the expression levels of JMJD2A. Flow cytometric (FCM) analysis and WST-8 assay were used to evaluate cell proliferation. Boyden chambers were used in cell migration and invasion assays to evaluate the cell exercise capacity. Expression levels of JMJD2A mRNA and protein in the siRNA group were both downregulated successfully by transfection. FCM results showed that the percentage of cells in the G0/G1 phase in the siRNA group was significantly greater than that in the blank (P<0.05) and negative control groups (P<0.05). Additionally, the mean absorbance in the siRNA group was significantly lower (P<0.05), as observed by WST-8 assay. Moreover, a decreased number of migrated cells in the siRNA group was observed (P<0.05) using a cell migration and invasion assay. These data indicated that knockdown of JMJD2A may cause inhibition of proliferation, migration and invasion of MCF-7 cells. This study provides a new perspective in understanding the molecular mechanisms underlying the progression of breast cancer and offers a potential therapeutic target for breast cancer.

## Introduction

Human breast cancer is the most frequently diagnosed cancer and the leading cause of cancer-related death among females. It was shown in the GLOBOCAN 2008 estimates that breast cancer accounted for 23% of total cancer cases and 14% of cancer mortalities (1). Due to the progression and metastasis of this tumor, the prognosis of patients with advanced stage breast cancer remains poor despite treatment with various therapy types. Better understanding of the molecular mechanisms underlying the progression of breast cancer is required for prevention and treatment.

Use of small interfering RNA (siRNA) to silence a target gene is a simple and effective method, particularly when specific siRNA is employed (2). By introducing double-stranded RNA homologous to a particular message and causing its sequence-specific degradation, RNA interference (RNAi) technique provides a rapid method with which to deplete mRNA as a post-transcriptional regulation.

Jumonji domain containing 2A (JMJD2A) was identified and characterized in 2004 (3). It is widely expressed in human tissues and cell lines. Moreover, the expression level of JMJD2A mRNA was found to be high in several cell types including prostate cancer, U2OS osteosarcoma, HT1376 bladder carcinoma and human T-cell lymphotropic virus 1-infected cell lines (4,5). JMJD2A belongs to the cancer-associated gene family of JMJD2 proteins (3), which, containing a JmjC domain, are lysine trimethyl-specific histone demethylases capable of catalyzing the demethylation of trimethylated H3K9 (H3K9me3) and H3K36 (H3K36me3) (6–8). However, the existing literature rarely pays close attention to the function of JMJD2A in breast cancer. It remains unclear whether the inhibition caused by knockdown of JMJD2A is a special case or a common phenomenon. Effects of knockdown of JMJD2A on other human breast cancer cell lines are unknown.

This study transfected chemically synthesized JMJD2A-specific siRNA into human breast cancer cell line MCF-7. The transcription level of JMJD2A mRNA, expression level of JMJD2A protein and the biological characteristics of MCF-7 cells, such as cell proliferation, migration and invasion ability, were investigated.

## Materials and methods

### JMJD2A siRNA synthesis

JMJD2A siRNA was chemically synthesized by Qiagen Technology Co., Ltd. (Shanghai, China). The sense sequence of the synthesized siRNA duplexes was: 5′-GAGUUAUCAACUCAAGAUA-3′, and its antisense sequence was: 5′-UAUCUUGAGUUGAUAACUC-3′. siRNA was diluted to 20 μmol/l with RNase-free water.

### Cell transfection

The MCF-7 human breast cancer cell line, which was preserved in our laboratory in logarithmic growth phase, was seeded into 6-well plates, at a density of 5×10^5^ cells per well and cultured in regular growth medium 24 h prior to transfection. Regular growth medium was replaced by serum-free Opti-MEM (Gibco, Invitrogen, Carlsbad, CA, USA) 8 h later. Transfection compounds were prepared in three groups as follows: siRNA group (100 μl of Opti-MEM, 6 μl of HiPerFect transfection reagent and 5 μl of JMJD2A siRNA), negative control group (100 μl of Opti-MEM, 6 μl of HiPerFect transfection reagent and 5 μl of negative control siRNA) and blank control group (100 μl of Opti-MEM). HiPerFect transfection reagent and negative control siRNA were purchased from Qiagen Technology Co. Ltd (Shanghai, China). Transfection compounds were placed at room temperature for 10 min and then dropped into 6-well plates, with 2200 μl of bulk volume per well. Both Opti-MEM and transfection compounds were replaced with complete medium 24 h after transfection. FAM-siRNA was transfected to measure the transfection efficiency simultaneously.

### Quantitative real-time PCR

Total RNA of the three groups was extracted with the RNAiso reagent kit (Takara, Dalian, China) 48 h after transfection. cDNA was generated by reverse transcription of 2 μg of total RNA using random primers and PrimeScript RT Master Mix (Perfect Real Time) (Takara) in a total reaction volume of 40 μl. The sequences of forward and the reverse oligonucleotide primers, specific to JMJD2A and housekeeping genes, were designed using Primer5 software. Primers (5′-CCAGAACCAACCAGGAGC-3′ and 5′-TTCACT GCGCGAGACCAT-3′ for JMJD2A; 5′-TGGCACCCAGCA CAATGAA-3′ and 5′-CTAAGTCATAGTCCGCCTAGAA GCA-3′ for β-actin) were synthesized by Shanghai Daweike Biotechnology Co. Ltd. (Shanghai, China).

### Real-time quantitative PCR was performed in an ABI PRISM 7500 real-time system

A 10-fold dilution of each cDNA was amplified in a 20-μl volume, using the SYBR Premix Ex Taq™ (Perfect Real Time) (Takara), at a final concentration of 0.2 μM for each primer. PCR cycle conditions were set as follows: 95°C for 30 sec, and 40 cycles of 95°C for 5 sec and 60°C for 34 sec. The amplification specificity was evaluated with melting curve analysis. Threshold cycle Ct, which correlates inversely with the target mRNA levels, was calculated using the second derivative maximum algorithm provided by the iCycler software. For JMJD2A, the mRNA levels were normalized to β-actin mRNA levels (9).

### Western blot analysis

Cells in different groups were homogenized 72 h after transfection in western blot analysis buffer containing 10 mM Tris-HCl (pH 7.4), 150 mM NaCl, 1% (v/v) Triton X-100, 1% sodium deoxycholate, 0.1% SDS, 5 mM EDTA, 1 mM PMSF, 0.28 kU/l aprotinin, 50 mg/l leupeptin, 1 mM benzamidine and 7 mg/l pepstain A. The homogenate was then centrifuged at 12,000 rpm for 10 min at 4°C and the supernatant was retained and preserved at −80°C for later use. Protein concentration was determined using a BCA kit (Pierce; Thermo Fisher Scientific, Rockford, IL, USA). Protein (20 μg) from each group was subject to electrophoresis on a 10% SDS-PAGE gel using a constant current. Proteins were transferred to nitrocellulose membranes on a semidry electrotransferring unit and incubated with monoclonal rabbit anti-human JMJD2A antibody (Cell Signaling Technology, Danvers, MA, USA; 1:1000) in Tris-buffered saline containing 0.1% Tween-20 (TBST) and 5% nonfat dry milk overnight at 4°C.

After overnight incubation with the primary antibodies, membranes were washed and incubated with HRP-labeled goat anti-rabbit secondary antibody (Santa Cruz Biotechnology Inc., Santa Cruz, CA, USA) in TBST for 2 h. Immunoreactivity was detected with enhanced chemiluminescent autoradiography (ECL kit, Amersham). The membranes were reprobed with β-actin (Cell Signaling Technology; 1:1000) after stripping. The signal intensity of primary antibody binding was quantitatively analyzed with Sigma Scan Pro 5 and was normalized to a loading control, β-actin (10).

### Flow cytometric anlysis (FCM)

Cells in different groups were collected with trypsinization 72 h after transfection and washed twice with PBS. Cells were fixed in 70% ethanol for 1 h at room temperature. After centrifugation, the cell pellet was resuspended in PBS (pH 7.4), containing 100 μl RNase A (1 mg/ml) and 400 μl propidium iodide (50 μg/ml). The cells were incubated for 30 min at room temperature, and the DNA content was determined by flow cytometry using a FACScan flow cytometer at 488 nm and the data were analyzed using Lightcycle software. The experiment was performed three times in triplicate (11). Proliferation indices (PI) were calculated as follows: PI = (S + G2/M)/(G0/G1 + S + G2/M) x 100%.

### WST-8 assay

MCF-7 cells were seeded into 96-well plates at a density of 5×10^3^ cells per well and cultured in regular growth medium 24 h before transfection. Regular growth medium was replaced by serum-free Opti-MEM 8 h later. These cells were grouped similarly to cell transfection. The bulk volume of the transfection compounds was 100 μl per well. Opti-MEM and transfection compounds were replaced by complete medium 24 h after transfection. After incubation for 72 h, MCF-7 cells were incubated for an additional 2 h with 20 μl of WST-8 dye (Beyotime Institute of Biotechnology, Haimen, China). Absorbance of the three groups was measured with a microplate reader (Model 550, Bio-Rad, Hercules, CA, USA) at a wavelength of 450 nm (A450). All experiments were carried out eight times (12).

### In vitro cell migration and invasion assay

The cells in different groups were treated with trypsin and re-suspended as single-cell solutions 24 h after transfection. A total of 2×10^5^ cells in 0.5 ml of serum-free RPMI 1640 medium were seeded on an 8-μm-pore polycarbonate membrane Boyden chamber insert in a Transwell^®^ apparatus (Costar, Cambridge, MA, USA), either coated with (invasion) or without (migration) Matrigel (BD Biosciences, San Jose, CA, USA). RPMI-1640 (600 μl) containing 20% FBS was added to the lower chamber. After incubation for 72 (invasion) or 36 h (migration) at 37°C in a 5% CO_2_ incubator, the cells on the top surface of the insert were removed with a cotton swab. The cells that migrated to the bottom surface of the insert were fixed in 100% methanol for 2 min, stained in Giemsa for 2 min, rinsed in PBS and then subjected to microscopic inspection (x200). Values for invasion and migration were obtained by counting the number of stained cells in five fields per membrane and data represented the average of three independent experiments (13).

### Statistical analysis

The data were presented as the means ± standard error (SE) for MCF-7 cells in each group. Statistical analysis was carried out by one-way ANOVA followed by Dunnett’s or Student’s t-test (two means comparison). Statistical analysis was carried out using the related programs in SPSS 12.0. Differences were considered to indicate statistical significance at P<0.05.

## Results

### JMJD2A siRNA synthesis

The sequence of chemically synthesized JMJD2A siRNA was consistent with the requirements, and the purity reached 98%. This met the experimental requirements.

### Observation of cell transfection results

**Figure 1:**
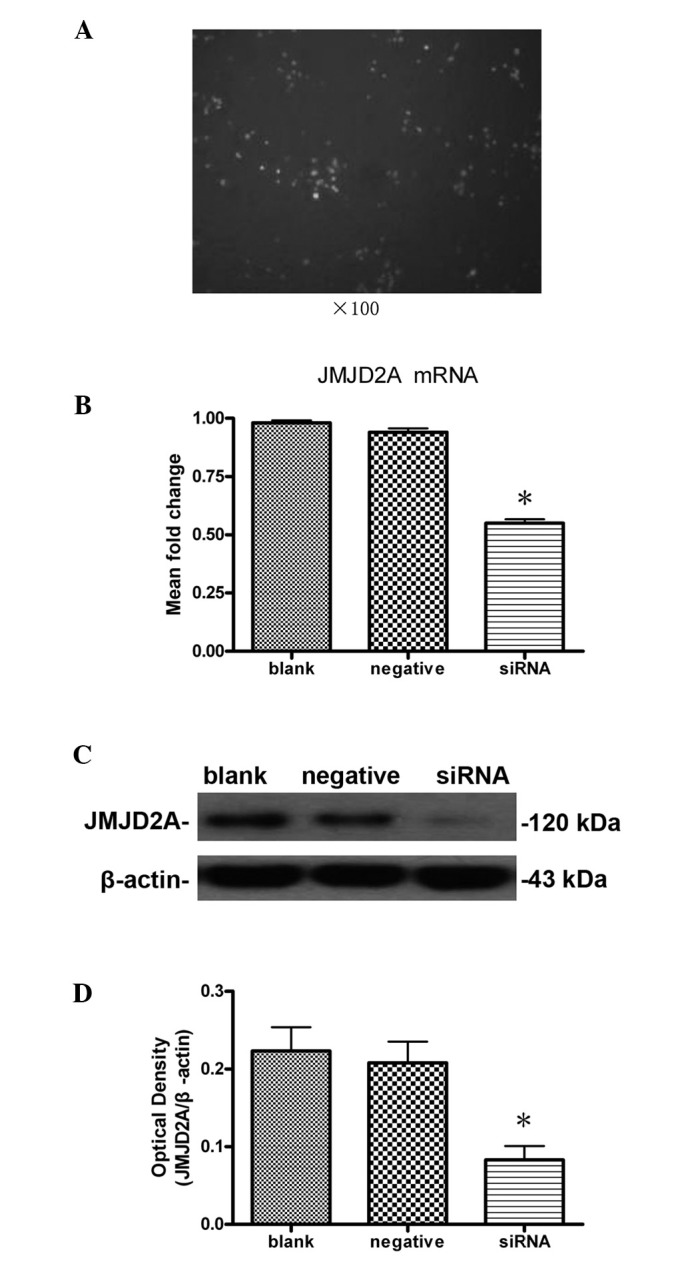
Transfection was successful and levels of JMJD2A mRNA and protein were both down-regulated. (A) Green fluorescent cells transfected with FAM-siRNA under fluorescence microscope. (B) Column diagram analysis for mRNA levels of JMJD2A. The JMJD2A-specific siRNA group exhibited a reduction in JMJD2A mRNA levels in MCF-7 cells. (C) Western blot analysis for JMJD2A protein. (D) Column diagram analysis for optical density by western blotting. JMJD2A protein levels were downregulated in the siRNA group. ^*^P<0.05 compared with blank and negative control groups.

MCF-7 cells transfected with FAM-siRNA were subjected to fluorescence microscopy 8 h after transfection. Cells that showed green fluorescence were considered to be successfully transfected. As shown in Fig. 1A, cell transfection was successful and HiPerFect transfection reagent was effective.

### Downregulation of JMJD2A mRNA levels by transfection with JMJD2A-specific siRNA

According to the results of quantitative real-time PCR (Fig. 1B), no significant difference (P>0.05) was detected in the levels of JMJD2A mRNA between the blank (0.98±0.02) and negative (0.94±0.03) control groups. The mRNA expression in the siRNA group (0.55±0.03) was significantly lower than that of the blank (P<0.05) and negative (P<0.05) control groups. These data indicate that JMJD2A mRNA levels in MCF-7 cells decreased significantly following transfection with JMJD2A siRNA. Transfection with JMJD2A-specific siRNA may result in JMJD2A mRNA degradation to silence the JMJD2A gene.

### Inhibition of JMJD2A protein expression in MCF-7 cells by transfection with JMJD2A-specific siRNA

Western blot analysis showed that the level of JMJD2A protein expression in the siRNA group (0.083±0.031) were significantly lower than those in the blank (0.223±0.053) and negative (0.208±0.047) control groups (P<0.05; Fig. 1C and D), whereas the difference between the blank and negative control groups was not significant (P>0.05; Fig. 1C and D). These data indicated that JMJD2A-specific siRNA silencing significantly reduced the levels of JMJD2A mRNA and protein expression in MCF-7 cells.

### Changes in cell cycle and inhibition of proliferation in MCF-7 cells due to knockdown of JMJD2A

**Figure 2:**
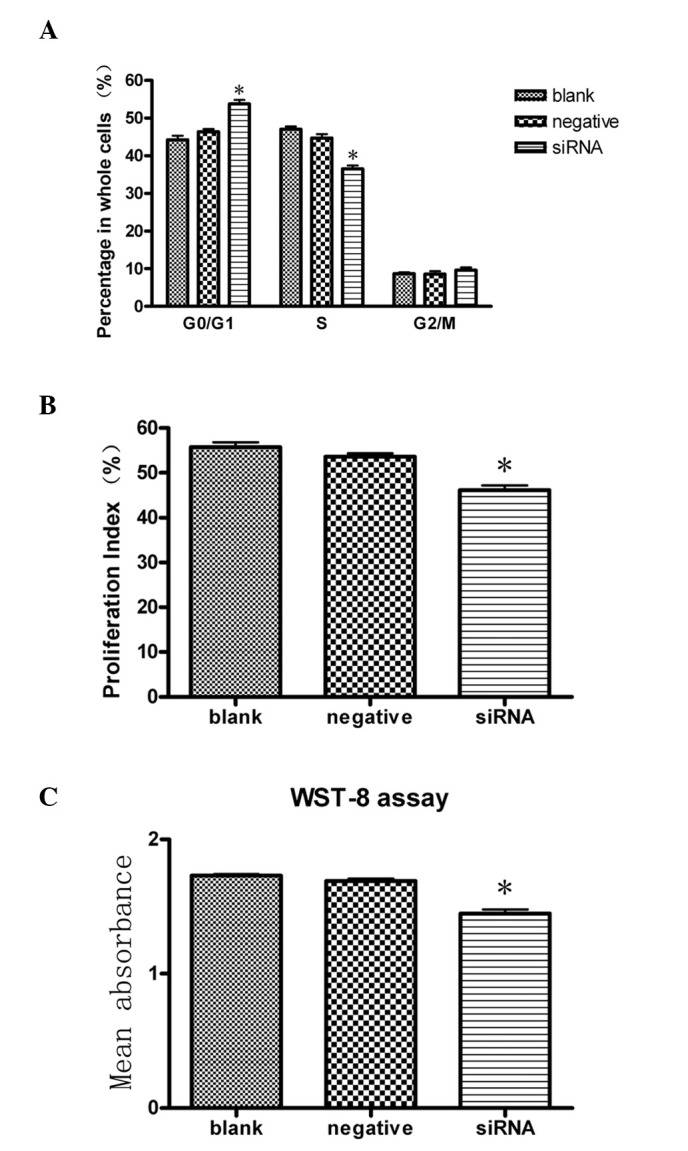
Knockdown of JMJD2A resulted in cell cycle change and proliferation inhibition. (A) Column diagram analysis of the percentages of cells at each phase of the cell cycle in three different cell groups: G0/G1 phase, S phase and G2/M phase. At G0/G1 phase, there was a significant difference in the percentage of cells in the siRNA group compared with the blank and negative control groups. At S phase, there was a significant difference in the percentage of cells in the siRNA group compared with the blank and negative control groups, whereas no significant differences were observed in the percentages of cells at G2/M phase in the three groups. (B) Column diagram analysis for the proliferation indices (PI) calculated in the three different cell groups. PI in the siRNA group was significantly lower than that in the blank and negative control groups. (C) Column diagram analysis for the mean absorbance of three different groups. The mean absorbance of the siRNA group was significantly lower than that of the blank and negative control groups. ^*^P<0.05, compared with the blank and negative control groups.

Cell cycle analysis by FCM revealed that JMJD2A siRNA was capable of inducing changes in the cell cycle of MCF-7 cells. The mean value of the experiments was shown in Fig. 2A and B. There were no significant differences (P>0.05) in the percentages of cells at each phase between the blank and negative control groups. Compared with the blank (44.24±1.86%) and negative (46.37±1.29%) control groups there was a significant difference (P<0.05) in the percentage of cells in the G0/G1 phase in the siRNA group (53.80±1.80%). Similarly, there was a significant difference (P<0.05) in the percentage of cells in the S phase in the siRNA group (36.55±1.52%) compared to the blank (47.06±1.26%) and negative (44.72±1.86%) control groups. However, there was no significant difference (P>0.05) in the percentage of cells in the G2/M phase in the siRNA group (9.64±1.26%), compared with blank (8.70±0.63%) and negative (8.58±1.35%) control group. Knockdown of JMJD2A was capable of increasing the percentage of cells in the G0/G1 phase and decreasing the percentage of cells in the S phase. The results indicate that the treatment was capable of arresting cells at the G1/S checkpoint and delaying the cell cycle into the S phase. Furthermore, proliferation indices (PI) of each group were calculated. We found that there was a significant difference (P<0.05) in the PI of the siRNA group (46.19±1.80%) versus those of the blank (55.76±1.86%) and negative (53.63±1.29%) control groups. Our results revealed changes in the cell cycle after transfection, indicating that cell proliferation was inhibited by transfection.

Additionally, the WST-8 assay was performed to test the effects of transfection with JMJD2A siRNA on the proliferation of MCF-7 cells in three different groups. As shown in Fig. 2C, there was no significant difference (P>0.05) in the average absorbance between the blank (1.73±0.02) and negative (1.69±0.03) control groups. The average absorbance in the siRNA group (1.45±0.05) was significantly lower than these values in the blank (P<0.05) and negative control groups (P<0.05). Absorbance is representative of the cell proliferation in WST-8 assay. The WST-8 assay results were consistent with the FCM results. These data indicated that transfection with JMJD2A siRNA significantly reduced the proliferation of MCF-7 cells.

### Suppression of MCF-7 cell migration and invasion in vitro by knockdown of JMJD2A

**Figure 3:**
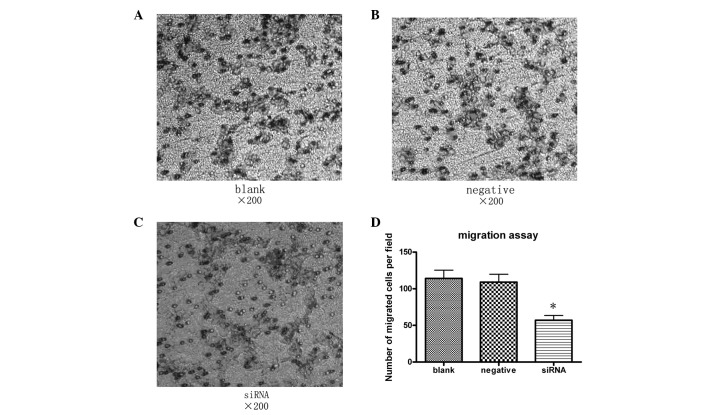
Knockdown of JMJD2A resulted in suppression of tumor cell migration. (A) Cells in the blank control group transversed the Transwell membrane. (B) Cells in the negative control group. (C) Cells in the siRNA group. (D) Column diagram analysis for the number of MCF-7 cells in the migration assay. The number of cells in the siRNA group (57±11.3) was decreased compared with these values in the blank (114±19.5) and negative control groups (109±18.7). ^*^P<0.05 compared with the blank and negative control groups.

**Figure 4:**
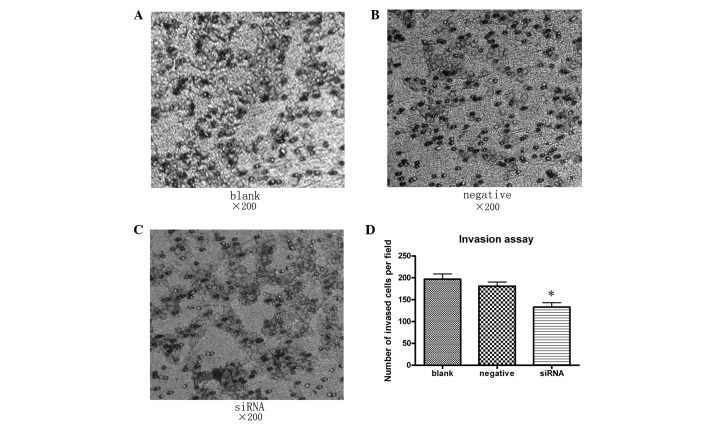
Knockdown of JMJD2A resulted in suppressing tumor cell invasion. (A) Cells in the blank control group transversed the Transwell membrane. (B) Cells in the negative control group. (C) Cells in the siRNA group. (D) Column diagram analysis for the number of MCF-7 cells in the invasion assay. The number of cells in the siRNA group (133±17.4) was decreased compared with these values in the blank (197±20.6) and negative control groups (181±16.3). ^*^P<0.05, compared with the blank and negative control groups.

As shown in Fig. 3, cell migration was significantly decreased in the siRNA group compared with the blank (P<0.05) and negative (P<0.05) control groups. Cells in the siRNA group showed significantly decreased invasiveness compared with the blank (P<0.05) and negative control groups (Fig. 4; P<0.05). These results demonstrated that transfection with JMJD2A siRNA reduced the migration and invasion of MCF-7 cells.

## Discussion

In this study, the results of the quantitative real-time PCR and Western blot analysis revealed that knockdown of JMJD2A in human breast cancer cell line MCF-7 was successful. Furthermore, knockdown of JMJD2A caused inhibition of proliferation, migration and invasion of MCF-7 cells. Human breast cancer is the leading cause of cancer-related death among females due to its powerful invasive ability and early metastatic potential. Understanding the pathological mechanism of breast cancer and identification of treatment target sites is a priority. This study provides a new perspective in understanding the molecular mechanisms underlying the progression of breast cancer, as well as identifies a potential therapeutic target in breast cancer.

Recent studies indicate that not only gene dysfunction but also histone modifications are involved in breast tumorigenesis (14). Coincidently, JMJD2A is a lysine trimethyl-specific histone demethylase with the capability of catalyzing the demethylation of H3K9me3 and H3K36me3 (6–8). H3K9 modifications are reported in numerous biological phenomena including germ cell development, X chromosome inactivation and DNA damage repair and apoptosis, and deregulated histone methylation is associated with tumorigenesis (15–17). Local chromatin architecture is strongly influenced by post-translational histone modifications such as methylation. Various histone modifications and histone variants combine to the proposition of the regulatory histone code. The histone code at least partly determines the transcriptional potential for a specific gene or a genomic region (18–20). H3K9me3 is predominant in coding regions of certain active genes and is enriched in heterochromatin, with a generally repressive nature (21–24). The intragenic permissive chromatin regions are flanked by H3K9me3 and the maintenance of the intragenic chromatin boundary appears to function as a checkpoint in elongation (25). The balance between methylated and demethylated histones may be broken by high expression of endogenous JMJD2A, which catalyzes demethylation of H3K9me3 excessively. Suv39H1, which is an H3K9 histone methyltransferase, functions as a tumor suppressor by maintaining H3K9 methylation levels (26,27). Based on the literature, we hypothesize that JMJD2A may take part in breast tumorigenesis through demethylation of H3K9me3 acting as transcriptional activators.

The inhibition due to knockdown of JMJD2A appears to support our hypothesis. However, the mechanism of JMJD2A in breast cancer is elusive. Here, we discuss two possible pathways involved according to the present literature.

One possible pathway is that JMJD2A may be involved in the estrogen signaling pathway. A recent study focused on JMJD2 family proteins, particularly JMJD2B, which is considered to have a similar function to that of JMJD2A in breast cancer. The study demonstrated that JMJD2B comprises a key component of the estrogen signaling pathway and the establishment of local epigenetic state and chromatin structure required for proper induction of ER-responsive genes (28). JMJD2B, which interacts with ERα and components of the SWI/SNF-B chromatin remodeling complex, was recruited to ERα target sites, demethylated H3K9me3 and facilitated transcription of ER-responsive oncogenes including MYB, MYC and CCND1, and knockdown of JMJD2B severely impaired estrogen-induced cell proliferation and, consequently, the tumor formation capacity of breast cancer cells. Our results are consistent with this. JMJD2A may have the same mechanism as JMJD2B.

The other possible candidate is the pRB-E2F complex pathway. Depletion of JMJD2A caused only a marginal defect in ER target gene induction in contrast to JMJD2B (28), which indicates another pathway in which JMJD2A may be involved. JMJD2A has molecular characterization in binding both retinoblastoma protein (pRB) and histone deacetylases (HDACs) (4). Associating with pRB, JMJD2A may recruit HDACs to the pRB-E2F complex, change the chromatin structure at the E2F-responsive promoters and induce repression transcription from E2F-dependent promoters (29,30). E2F1, 4 and their complexes with HDAC play an important role in down-regulating the expression of the maternally imprinted tumor suppressor gene ARHI in breast cancer cells (31). Expression of ARHI is markedly downregulated in breast cancer, and reactivation of ARHI expression in breast cancer cells is associated with decreased H3K9me3, which is demethylated by JMJD2A (32). These studies support the possibility of the pRB-E2F complex pathway.

Participating in either the estrogen signaling pathway or pRB-E2F complex pathway or both, JMJD2A may play a diverse role in human breast cancer. Though the exact mechanism remains unclear, inhibition effects from knockdown of JMJD2A indicate that JMJD2A participates in human breast cancer and may be a potential therapeutic target.

To date, there are few studies focusing on JMJD2A in human breast cancer. Our research verified the inhibition effects. JMJD2A may be a potential therapeutic target in human breast cancer. However, the present results were based on studies in a single cell line *in vitro*. They have merely exposed the ‘tip of the iceberg’ of the role of JMJD2A in human breast cancer. Further studies to elucidate the pleiotropic functions of JMJD2A and its contribution to human breast cancer *in vitro* and *in vivo* are required.
